# Negative magnetostrictive paper formed by dispersing CoFe_2_O_4_ particles in cellulose nanofibrils

**DOI:** 10.1038/s41598-023-31655-z

**Published:** 2023-04-15

**Authors:** Takumi Keino, Lovisa Rova, Alia Gallet--Pandellé, Hiroki Kurita, Fumio Narita

**Affiliations:** 1 Department of Frontier Sciences for Advanced Environment, Graduate School of Environmental Studies, Tohoku University, Sendai, Japan; 2 Ångström Laboratory, Disciplinary Domain of Science and Technology, Department of Chemistry, Uppsala University, Uppsala, Sweden; 3 Department of Materials Science and Engineering, INSA-Lyon, Université de Lyon, Villeurbanne Cedex, France

**Keywords:** Mechanical engineering, Materials for energy and catalysis, Soft materials

## Abstract

Polymers are often combined with magnetostrictive materials to enhance their toughness. This study reports a cellulose nanofibril (CNF)-based composite paper containing dispersed CoFe_2_O_4_ particles (CNF–CoFe_2_O_4_). Besides imparting magnetization and magnetostriction, the incorporation of CoFe_2_O_4_ particles decreased the ultimate tensile strength and increased the fracture elongation of the CNF–CoFe_2_O_4_ composite paper. CNF was responsible for the tensile properties of CNF–CoFe_2_O_4_ composite paper. Consequently, the magnetic and magnetostrictive properties and tensile properties of CNF–CoFe_2_O_4_ composite paper can be controlled by changing the mixture ratio of CNF and CoFe_2_O_4_ particles.

## Introduction

To alleviate the global energy crisis and environmental pollution, many researchers are exploring alternative energy technologies that harvest energy from the ambient environment (e.g., mechanical vibrations)^1–3^. When the ambient energy supply is limited, piezoelectric-energy harvesting devices generate sufficient power for targeted devices such as Internet of Things sensors^4^. For this purpose, piezoelectric materials, composites, and devices have been actively researched^5–11^ and their vibration-energy harvesting performances have been evaluated.

Magnetostrictive materials can deform under an external magnetic field^12^. The magnetostrictive effect was first described by James Prescott Joule in 1842^13^. He reported that iron, a ferromagnetic material, changes its dimension in response to a magnetic field. Since that time, researchers have developed various magnetostrictive materials such as Tb–Dy–Fe alloys (terfenol-D), Fe–Ga alloys (galfenol), Fe–Co alloys, and CoFe_2_O_4_ (cobalt ferrites)^14–18^. Magnetostrictive materials, composites, and devices are also attracting attention in the energy harvesting field^19–24^. Terfenol-D and galfenol are well-known giant magnetostrictive alloys showing good magnetostrictive properties at room temperature, but are brittle and expensive^1,16^.

To overcome the brittleness of magnetostrictive materials, many researchers have dispersed magnetostrictive particles through a polymer matrix, forming magnetostrictive polymer composites (MPCs)^25^. Under an external magnetic field, the magnetostrictive particles deform and exert a force on the polymer matrix, deforming the whole composite. Equilibrium is achieved by balancing the stresses generated in the magnetostrictive particles and the polymer matrix, resulting in overall deformation of the MPC. MPCs are potentially applicable to current and stress sensing, vibration damping, actuation, health monitoring, and biomedical applications. In addition, they are easier to manufacture to the required geometry than the above-mentioned giant magnetostrictive alloys. Previous studies on MPCs have reported terfenol-D particles^26^ and galfenol particles^27^ dispersed through an epoxy resin matrix (terfenol-D/epoxy and galfenol/epoxy composites, respectively), Fe–Co alloy particles dispersed through a polyurethane matrix (Fe–Co/PU composites)^28^ and various others^29,30^. Positive magnetostriction values of 1600, 360, and 70 ppm have been reported in terfenol-D/epoxy, galfenol/epoxy, and Fe–Co/PU, respectively. However, MPCs with negative magnetostrictive effect have only been investigated to a small degree. Nersessian et al.^31^ reported saturation magnetostrictions of − 24 and − 28 ppm in hollow and solid nickel composites, respectively. Similarly, Ren et al.^32^ reported negative magnetostriction in polymer-bonded Sm_0.88_Dy_0.12_Fe_1.93_ pseudo-1-3 composites.

Recently, paper- and cellulose-based devices have gained increasing attention^33^ because paper is low cost (~ 0.005 $/m^2^), biocompatible, environmentally friendly, 100% recyclable, and more stretchable than other polymer-based flexible devices^34^. Cellulose fiber is inexpensive, bio-based, biodegradable, non-hazardous, recyclable, and low-density^35^. Cellulose nanofibrils (CNFs) in particular show outstanding strength, stiffness, and toughness^36^ and are expected to be utilized as reinforcing fibers^37–43^.

Mattos et al.^44^ showed that nanonetworks created from CNFs can form superstructures with virtually any kind of particle because of supramolecular cohesion provided by the fibrils. This cohesion was shown to be a result of the high aspect ratio of the fibrils. Yermakov et al.^45^ fabricated magnetostrictive nanocellulose membranes by embedding terfenol-D particles into CNFs. After evaluating the magnetostrictive properties of the membranes, they found that various orientations of the terfenol-D particles were induced in the membranes, and that particles with in-plane alignment showed the strongest magnetostrictive effect. Kim et al.^46^ fabricated a magnetostrictive actuator string which could respond to an external magnetic field, by combining magnetic nanoparticles of Fe_2_O_3_ in a CNF matrix. However, there are no publications where magnetostrictive composites were made by combining CNFs and CoFe_2_O_4_. Antonel et al.^47^ produced a CoFe_2_O_4_-poly(aniline) composite by embedding CoFe_2_O_4_ nanoparticles inside a poly(aniline) polymer matrix. They showed that due to particle-polymer interactions, by varying the particle-polymer ratio one can modulate the magnetic behavior of the material.

In the present study, CoFe_2_O_4_ particles were dispersed through CNF to form CNF–CoFe_2_O_4_ composite papers. This paper reports the magnetic, magnetostrictive, and tensile properties of the papers. The microstructures of the CNF–CoFe_2_O_4_ composite papers were observed using a scanning electron microscope (SEM) and an X-ray diffraction (XRD) system.

## Experimental procedure

### Specimen preparation

**Figure 1:**
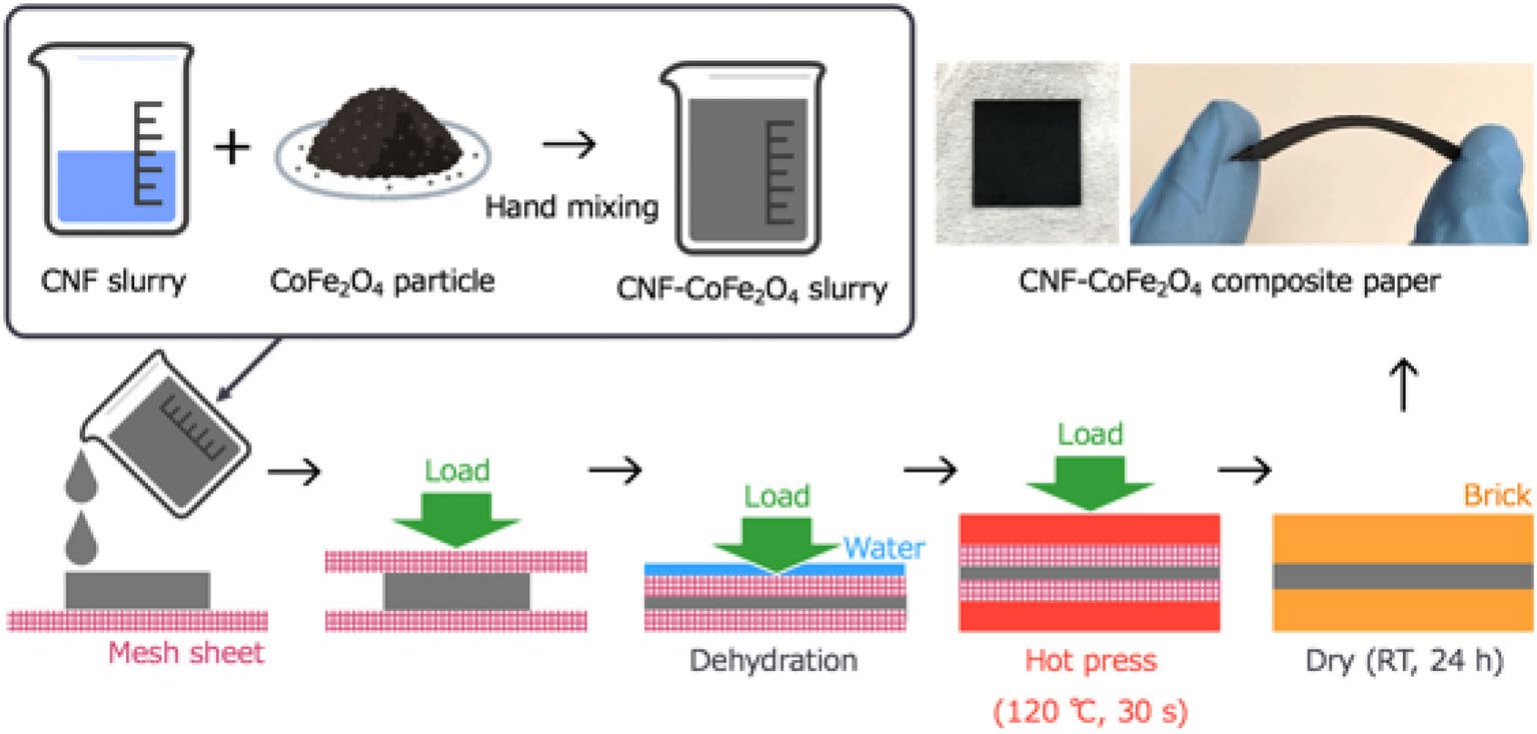
Schematic of the fabrication process of the CNF–CoFe_2_O_4_ composite papers (drew by Microsoft PowerPoint for Mac, version 16.70).

Figure 1 is a schematic of the fabrication process of the CNF–CoFe_2_O_4_ composite papers. The starting materials were CoFe_2_O_4_ particles (Kojundo Chemical Laboratory Co., Ltd., Japan) and a 2 wt% CNF slurry (IMA-10002, Sugino Machine, Japan). The CoFe_2_O_4_ particle size distribution was measured by a laser diffraction particle size analyzer (MASTERSIZER 3000, Malvern Panalytical, Spectris, UK). The CoFe_2_O_4_ particles and CNF slurry were manually mixed for 5 min at room temperature by hand-mixing. Using different weight ratios of CoFe_2_O_4_ particles: CNF slurry, 3 solutions were prepared: 5:95, 20:80 and 35:65, total weight 20 g. The solutions were sandwiched between two mesh sheets sized 100 $\times$ 100 mm^2^. The samples were compressed and dehydrated under an ultra-compact manual hydraulic heating press (Model IMC-195A-E, Imoto Mfg. Co., Ltd., Japan) operated at 120 °C for 30 s. After peeling off the mesh sheets, the dehydrated CNF–CoFe_2_O_4_ composite papers were further compressed and dried using a 2500 g weight for 24 h. The circular CoFe_2_O_4_ plate ($\phi$ 15 $\times$ 2.25 mm^3^) was then consolidated by spark plasma sintering (SPS, SPS-1050, Fuji Electric Industrial Co., Ltd., Japan), under a compressive stress of 20 MPa at 1000 °C for 10 min in a vacuum. A reference CoFe_2_O_4_ plate was cut to a size of 10 $\times$ 10 $\times$ 2.25 mm^3^ and reserved for further measurements.

### Density

To obtain the densities of the CoFe_2_O_4_ composite papers and CoFe_2_O_4_ sintered plate, the lengths and thicknesses of the samples were measured using an electronic digital caliper (SDV-150, Fujiwara Industrial Co., Ltd., Japan) and a digital thickness gauge (MDC-SX, Mitutoyo, Japan), respectively, and the weights of the samples were measured on a digital scale (ALE223R, Sinko Denshi Co., Ltd., Japan).

### Microstructure analysis

The microstructures of the CNF–CoFe_2_O_4_ composite papers and CoFe_2_O_4_ particles were investigated in a multipurpose XRD system (Ultima IV, Rigaku Co., Japan). The XRD patterns were obtained under CuK${\upalpha }$ radiation with a counting time of 1.67/s, a step size of 0.02°, a voltage of 40 kV, and a current of 40 mA. The scanning range was determined to be between 10° and 70°. The microstructures of the CNF–CoFe_2_O_4_ composite papers were observed using a field emission SEM (FE-SEM) (SU-70, Hitachi High-Tech Co., Ltd., Japan), with an acceleration voltage of 5 kV and a working distance of 10 mm. In preparation for FE-SEM, the surfaces of the CNF–CoFe_2_O_4_ composite papers were sputtered for 30 s using an ion sputtering device (E-1045, Hitachi High-Tech Co., Ltd., Japan) with a discharge current of 15 mA at 15 Pa to provide electrical conductivity to the composite papers. Furthermore, the FE-SEM was equipped with an energy dispersed X-ray spectrometer (EDX) for measuring the carbon (C), oxygen (O), cobalt (Co) and iron (Fe) concentrations in the CNF–CoFe_2_O_4_ composite papers. The EDX was operated at an acceleration voltage of 5 kV and a working distance of 15 mm.

### Magnetic and magnetostrictive properties

The magnetic properties of the CNF–CoFe_2_O_4_ composite papers were evaluated using a vibrating sample magnetometer (VSM) (BHV-50H, Riken Denshi Co., Ltd., Japan) calibrated to 4.931 emu. The VSM was calibrated on a pure nickel plate sized 10 $\times$ 10 $\times$ 0.1 mm^3^. The applied magnetic field range was ± 759 kA/m. The magnetostrictive properties of the CNF–CoFe_2_O_4_ composite papers were evaluated under the VSM electromagnets. The electromagnets were separated by 50 mm. The applied magnetic field was measured with a gauss meter (GM-4002, Denshijiki Industry Co., Ltd., Japan). The strains of the CNF–CoFe_2_O_4_ composite papers were measured by an orthogonal strain gauge (JFGS-1-120-D16-16 L3M2S, Kyowa Electronic Instruments Co., Ltd., Japan) positioned on the sample surface under an applied magnetic field range of ± 733 kA/m. The data were collected by data loggers (NR-ST04 and NR-HA08, Keyence, Japan)^28^.

### Tensile properties

The tensile properties of CNF–CoFe_2_O_4_ composite paper specimens sized 30 $\times$ 15 mm^2^ were investigated on a compact tabletop tester (EZ-SX, Shimazu Co., Ltd., Japan) with a 500-N load cell (Shimazu Co., Ltd., Japan). The tensile grips were separated by 15 mm. #600-grit sandpaper was inserted between the CNF–CoFe_2_O_4_ composite paper and the tensile grips to prevent slipping.

## Theory

This section formulates the problem of predicting the effective magneto-mechanical properties of the CNF–CoFe_2_O_4_ composite paper. In rectangular Cartesian coordinates *x*_*i*_ (O-*x*_1_, *x*_2_, *x*_3_), the constitutive equations of the heterogeneous magnetostrictive composite material are given by Eqs. (1) and (2)

1 $$\langle{\varepsilon }_{ij}\rangle={s}_{ijkl}^{*\mathrm{H}}{\langle\sigma }_{kl}\rangle+{d}_{kij}^{*}\langle{H}_{k}\rangle$$

2 $$\langle{B}_{i}\rangle={d}_{ikl}^{*}\langle{\sigma }_{kl}\rangle+{\mu }_{ik}^{*\mathrm{T}}\langle{H}_{k}\rangle$$

where $\langle{\varepsilon }_{ij}\rangle,\langle{\sigma }_{kl}\rangle,\langle{B}_{i}\rangle$ and $\langle{H}_{k}\rangle$ are the average components of the strain tensor, stress tensor, magnetic flux density vector, and magnetic field intensity vector, respectively, and ${s}_{ijkl}^{*\mathrm{H}}$, ${d}_{kij}^{*}$ and ${\mu }_{ik}^{*\mathrm{T}}$ are the elastic compliance under a constant magnetic field, the piezomagnetic constant, and the magnetic permittivity under a constant stress, respectively. Hereafter, the asterisk (*) will denote the effective average properties of the magnetostrictive composite material. This formulation adopts the compressed matrix notation, which is more useful than the extended tensor notation when discussing symmetry. In this matrix notation, *ij* or *kl* (*i*, *j*, *k*, *l* = 1, 2, 3) is replaced with *p*, *q* (valued from 1 to 6). Equations (1) and (2) are then rewritten as Eqs. (3) and (4)

3 $$\langle{\varepsilon}_{p}\rangle={s}_{pq}^{*\mathrm{H}}\langle{\sigma }_{q}\rangle+{d}_{kp}^{*}\langle{H}_{k}\rangle$$

4 $$\langle{B}_{i}\rangle={d}_{iq}^{*}\langle{\sigma }_{q}\rangle+{\mu }_{ik}^{*\mathrm{T}}\langle{H}_{k}\rangle$$

To demonstrate the effective properties of the CNF–CoFe_2_O_4_ composite papers, we consider the longitudinal magnetostrictive effect, meaning that the external field (either mechanical stress or a magnetic field) acts along the *x*_3_-direction (the easy magnetization axis of the composite paper). The magneto-mechanical coupling factor is given by Eq. (5)

5 $${k}_{33}^{2}=\frac{{d}_{33}^{*2}}{{s}_{33}^{*\mathrm{H}}{\mu }_{33}^{*\mathrm{T}}}=\frac{{E}^{*}{d}_{33}^{*2}}{{\mu }_{33}^{*\mathrm{T}}}$$

where ${E}^{*}$ is the Young’s modulus (slope of the stress–strain plot) of the CNF–CoFe_2_O_4_ composite paper. Note that the material properties ${E}^{*}$, ${d}_{33}^{*}$ and ${\mu }_{33}^{*\mathrm{T}}$ are functions of the volume fractions of the CoFe_2_O_4_ particles (${V}_{\mathrm{cfo}}$), $\mathrm{the}$ pores (${V}_{\mathrm{p}}$), and of the CNF matrix $({V}_{\mathrm{m}} = 1-({V}_{\mathrm{cfo}}+{V}_{\mathrm{p}}))$, respectively. Thus the material properties are calculated by inserting the volume fractions into Eqs. (6)–(8):

6 $${E}^{*}={E}^{*}\left({\mathrm{V}}_{\mathrm{cfo}}, {V}_{\mathrm{p}},{V}_{\mathrm{m}}\right)$$

7 $${d}_{33}^{*}={d}_{33}^{*}\left({V}_{\mathrm{cfo}}, {V}_{\mathrm{p}},{V}_{\mathrm{m}}\right)$$

8 $${\mu }_{33}^{*\mathrm{T}}={\mu }_{33}^{*\mathrm{T}}\left({V}_{\mathrm{cfo}}, {V}_{\mathrm{p}},{V}_{\mathrm{m}}\right)$$

where $\mathrm{the}$ subscripts cfo, p, and m represent the CoFe_2_O_4_ particles, pores, and matrix (i.e., CNF), respectively.

## Results and discussion

The weight fractions of CoFe_2_O_4_ (${W}_{\mathrm{cfo}}$) and CNF (${W}_{\mathrm{m}}$) in the composite papers are displayed in Table 1. The real densities of the CNF–CoFe_2_O_4_ composite papers were 0.7983 g/cm^3^ (sample from 5:95 solution), 1.1967 g/cm^3^ (sample from 20:80 solution), and 1.5058 g/cm^3^ (sample from 35:65 solution). Assuming that all water was evaporated from the dried CNF–CoFe_2_O_4_ composite paper and taking the theoretical densities of CoFe_2_O_4_ and cellulose (5.29^48^ and 1.50 g/cm^3^, respectively), the volume fractions of CoFe_2_O_4_ particles in samples made from the 5:95, 20:80, and 35:65 solutions were calculated as 10.9, 21.0, and 27.5 vol%, respectively, in the final CNF–CoFe_2_O_4_ composite papers (see Table 1). The average thicknesses of the CNF–CoFe_2_O_4_ composite papers containing 10.9, 21.0, and 27.5 vol% CoFe_2_O_4_ were 0.25, 0.49, and 0.92 mm, respectively. The real density of the CoFe_2_O_4_ sintered plate was 4.298 g/cm^3^. The relative density of the CoFe_2_O_4_ sintered plate was calculated as 81.2% of the theoretical density of 5.29 g/cm^3^.

**Table 1:** Parametric values of the CNF–CoFe_2_O_4_ composite papers.

CoFe_2_O_4_ content	10.9 vol%	21.0 vol%	27.5 vol%
${W}_{\mathrm{cfo}}$	72.46	92.59	96.42
${W}_{\mathrm{m}}$	27.54	7.41	3.58
${V}_{\mathrm{cfo}}$	0.109	0.210	0.275
${V}_{\mathrm{p}}$	0.744	0.731	0.689
${V}_{\mathrm{m}}$	0.147	0.059	0.036
Apparent ${E}^{*}$ [GPa]	0.523	0.269	0.195
${d}_{33}^{*}$ [m/A]	− 8.95 × 10^−12^	− 66.5 × 10^−12^	− 166 × 10^−12^
$\mathrm{Apparent} \,{\mu }_{33}^{*\mathrm{T}}$[H/m]	0.0769 × 10^−6^	0.127 × 10^−6^	0.228 × 10^−6^
Apparent ${k}_{33}^{2}$	5.45 × 10^−16^	9.37 × 10^−15^	2.36 × 10^−14^

**Figure 2:**
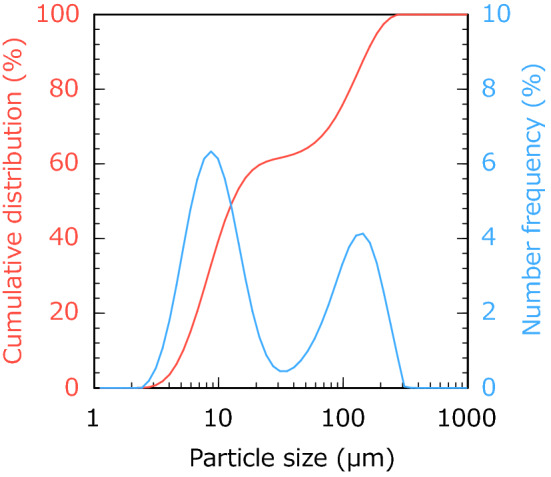
Size distribution of the CoFe_2_O_4_ particles.

**Figure 3:**
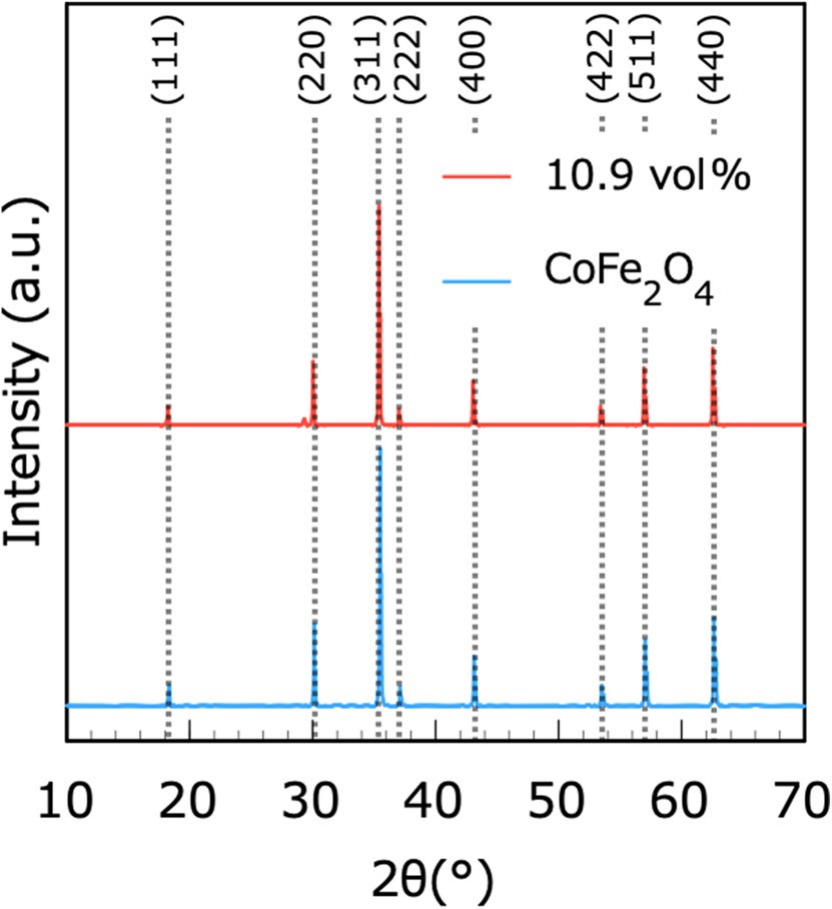
XRDs patterns of the 10.9 vol% CNF–CoFe_2_O_4_ composite paper (red) and CoFe_2_O_4_ particles (blue).

**Figure 4:**
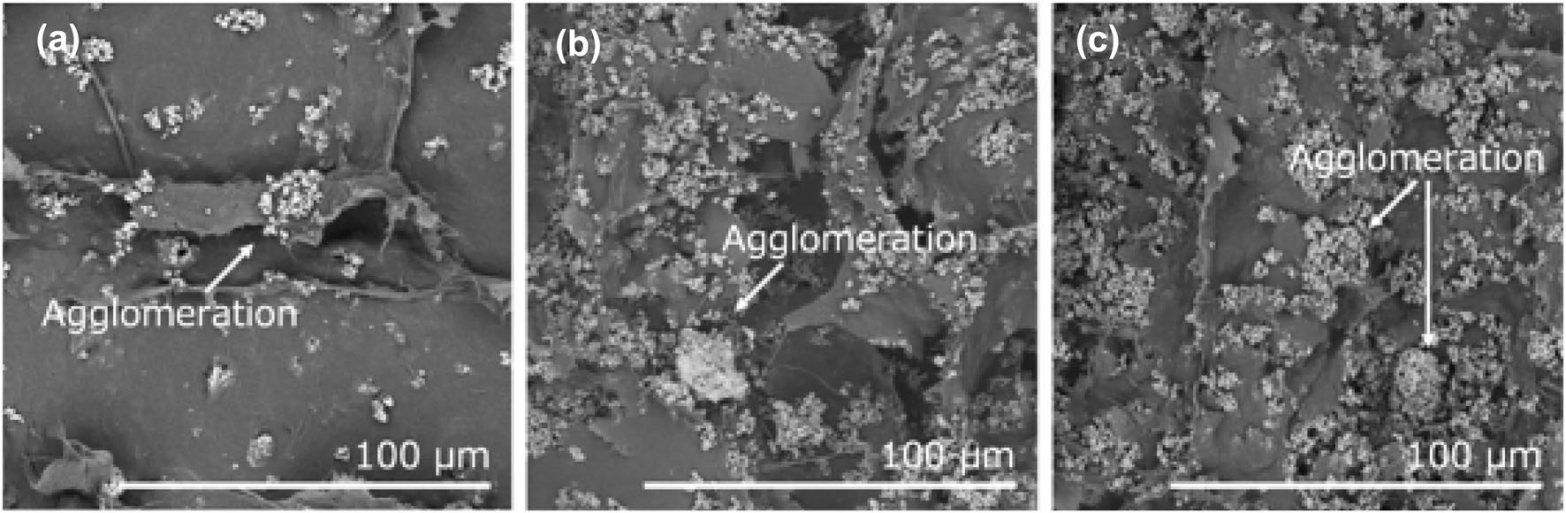
SEM images of the CNF–CoFe_2_O_4_ composite papers with different CoFe_2_O_4_ contents: (**a**) 10.9, (**b**) 21.0 and (**c**) 27.5 vol%.

**Figure 5:**
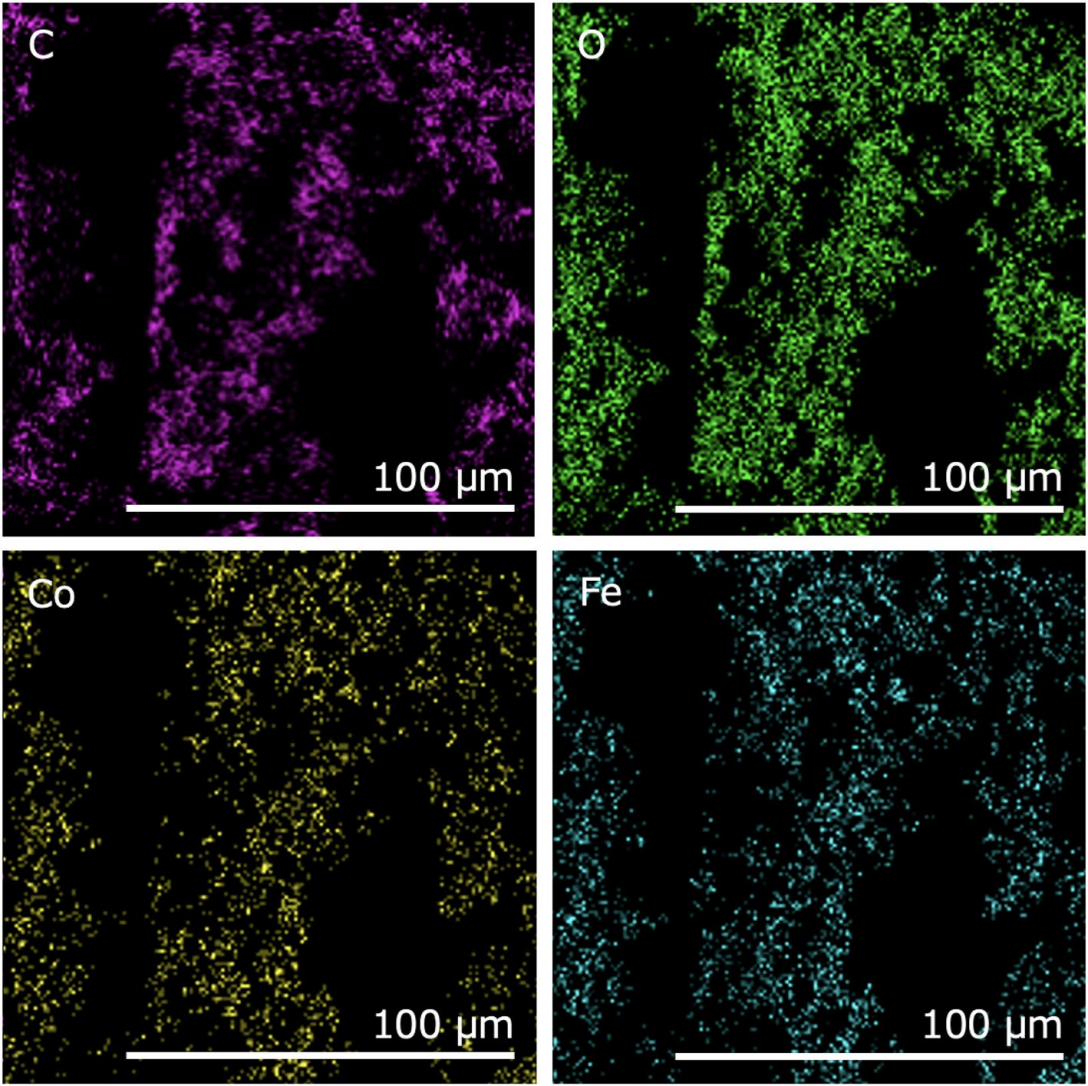
EDS mappings of the 27.5 vol% CNF–CoFe_2_O_4_ composite paper with 27.5 vol% CoFe_2_O_4_ content.

Figure 2 shows the size distributions of the CoFe_2_O_4_ particles. The CoFe_2_O_4_ powder comprised small and large particle populations with approximate diameters of 10 and 150 μm, respectively. As shown in Fig. 3, the XRD patterns of the 10.9 vol% CNF–CoFe_2_O_4_ composite paper matched those of the CoFe_2_O_4_ particles. Therefore, the CoFe_2_O_4_ was stable against chemical transformations during the fabrication process. Figure 4 shows the SEM images of the CNF–CoFe_2_O_4_ composite papers. Although the CoFe_2_O_4_ particles were dispersed through the CNF matrix, they were agglomerated by the hand mixing process. The size distribution peak at 150 μm in Fig. 2 was probably contributed by large agglomerates of CoFe_2_O_4_ particles. Figure 5 shows the EDX mapping of the 27.5 vol% CNF–CoFe_2_O_4_ composite paper (the micrograph is shown in Fig. 4c). The EDX detected C, O, Co, and Fe on the surface of the CNF–CoFe_2_O_4_ composite papers, implying that CoFe_2_O_4_ was stable against chemical reactions during the fabrication process, reconfirming the XRD results. It should be noted that no characteristic intensities of the X-ray appeared in the shadow of the EDX detector because they were attenuated by the uneven surface of the CNF–CoFe_2_O_4_ composite paper surface.

**Figure 6:**
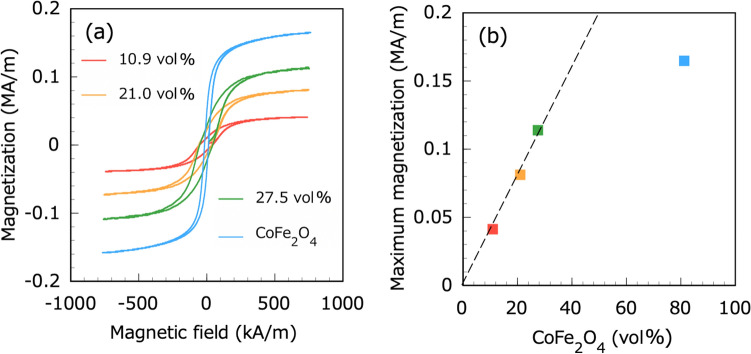
Magnetic properties of the CNF–CoFe_2_O_4_ composite papers and CoFe_2_O_4_ plate: (**a**) magnetization versus magnetic field curve and (**b**) plot of maximum magnetization versus CoFe_2_O_4_ volume fraction.

Figure 6 shows the magnetic properties of the CNF–CoFe_2_O_4_ composite papers and CoFe_2_O_4_ plate. The CoFe_2_O_4_ additives magnetized the CNF paper. The maximum magnetization of the CNF–CoFe_2_O_4_ composite paper linearly increased with increasing proportion of CoFe_2_O_4_ particles. Consistent with the present results, Williams et al.^49^ reported that the magnetic properties of magnetizing cellulose fibers depend on the volume percentage of the implemented magnetic filler in the fiber network. The magnetic curve of the CNF–CoFe_2_O_4_ composite paper reached saturation more slowly than the CoFe_2_O_4_ sintered plate. In Eq. (4), the effective magnetic permittivity ${\mu }_{33}^{*\mathrm{T}}$ of the CNF–CoFe_2_O_4_ composite paper under stress-free conditions was given by Eq. (9)

9 $${\mu }_{33}^{*\mathrm{T}}=\frac{{B}_{3}}{{H}_{3}}$$

The apparent effective magnetic permittivities ${\mu }_{33}^{*\mathrm{T}}$ of CNF–CoFe_2_O_4_ with CoFe_2_O_4_ contents of 10.9, 21.0, and 27.5 vol% were evaluated as 0.0769 $\times$ 10^−6^, 0.127 $\times$ 10^−6^, and 0.228 $\times$ 10^−6^ H/m, from the initial slope in Fig. 6a (see Table 1).

**Figure 7:**
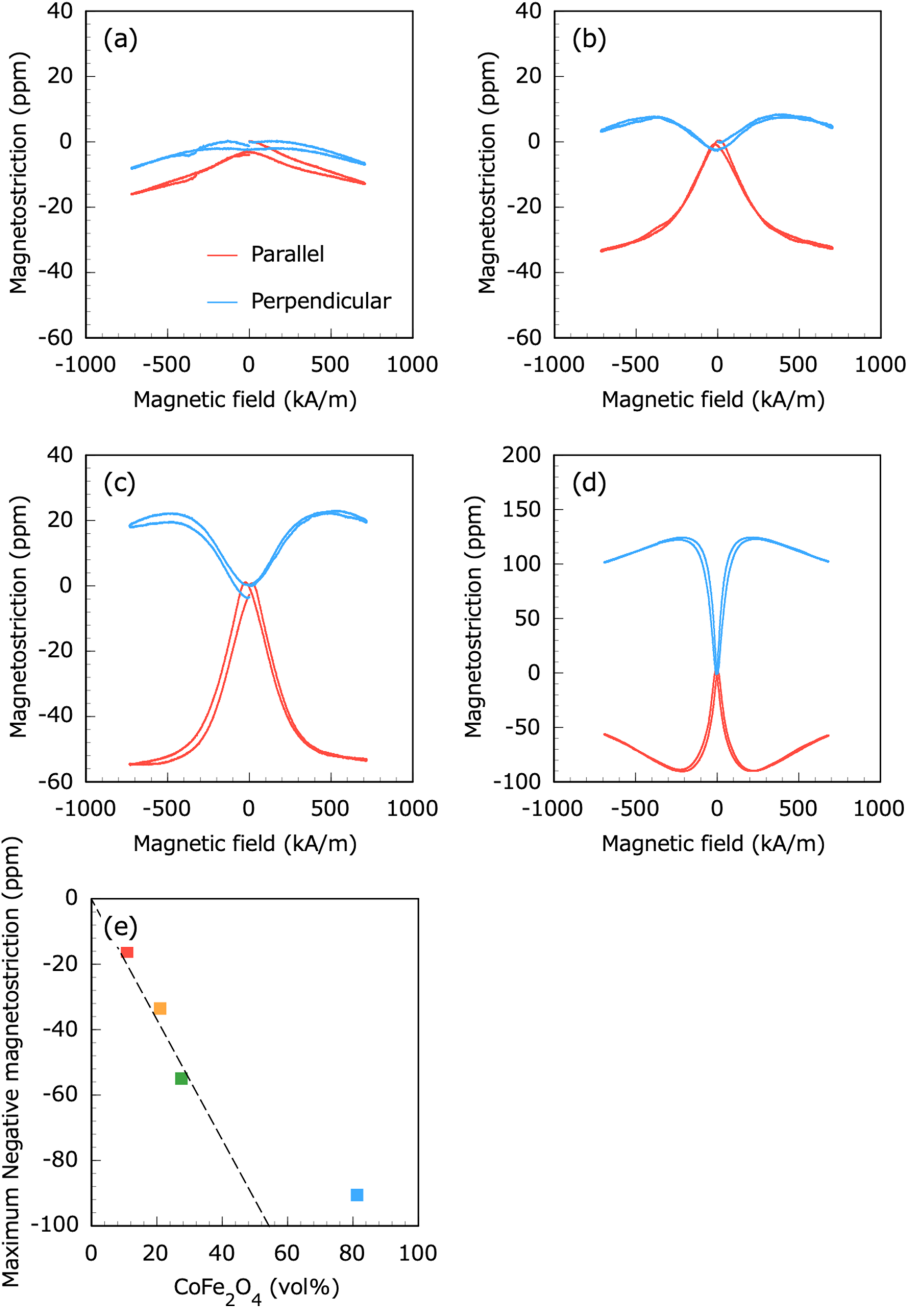
Magnetostrictive properties of the CNF–CoFe_2_O_4_ composite papers with CoFe_2_O_4_ contents of (**a**) 10.9, (**b**) 21.0 and (**c**) 27.5 vol% and (**d**) the CoFe_2_O_4_ plate; (**e**) plot of maximum negative magnetostriction versus CoFe_2_O_4_ volume fraction.

Figure 7 shows the magnetostrictive properties of the CNF–CoFe_2_O_4_ composite papers and CoFe_2_O_4_ plate. In the CNF–CoFe_2_O_4_ composite paper, the magnetostriction was negative and positive in the directions parallel and perpendicular to the magnetic field, respectively, as expected. The magnetostriction of CoFe_2_O_4_ plate first increased to a maximum negative value and then decreased. The maximum negative value of the CoFe_2_O_4_ plate was − 90 ppm under a magnetic field of 217 kA/m. Bozorth et al.^50^ said that CoFe_2_O_4_ has two magnetostriction coefficients ${\lambda }_{100}$ and ${\lambda }_{111}$: ${\lambda }_{100}<0$ and ${\lambda }_{111}>0$ at 300 K. Since the easy magnetization axis of CoFe_2_O_4_ is [100], correspondingly, it has a large negative ${\lambda }_{100}$ and a small positive ${\lambda }_{111}$^51,52^. It is believed that the same phenomenon occurred. The maximum negative magnetostriction of the CNF–CoFe_2_O_4_ composite paper deviated from the fitting line (see Fig. 7e). It should be noted that the 10.9 and 21.0 vol% CNF–CoFe_2_O_4_ composite papers failed to achieve magnetostrictive saturation under a magnetic field of ${H}_{3}=\pm$ 733 kA/m. These results imply that the CNFs between the CoFe_2_O_4_ particles deformed with magnetostriction of the CoFe_2_O_4_ particles and facilitated linear magnetostriction of the whole CNF–CoFe_2_O_4_ composite paper. In Eq. (3), the effective piezomagnetic constant ${d}_{33}^{*}$ of the CNF–CoFe_2_O_4_ composite paper under stress-free conditions was calculated as Eq. (10).

10 $${d}_{33}^{*}=\frac{{\varepsilon }_{3}}{{H}_{3}}$$

After straight-line fitting of the linear portions of the curves in Fig. 7a–c, the ${d}_{33}^{*}$ values of the CNF–CoFe_2_O_4_ composite papers containing 10.9, 21.0, and 27.5 vol% CoFe_2_O_4_ were calculated as − 8.95 $\times$ 10^−12^, − 66.5 $\times$ 10^−12^, and − 166 $\times$ 10^−12^ m/A, respectively (see Table 1). Clearly, the ${d}_{33}^{*}$ of the CNF–CoFe_2_O_4_ composite paper increased with increasing CoFe_2_O_4_ particle addition. Using Eq. (3), the effective piezomagnetic constant ${d}_{31}^{*}$ of the CNF–CoFe_2_O_4_ composite paper under stress-free conditions was obtained as Eq. (11).

11 $${d}_{31}^{*}=\frac{{\varepsilon }_{1}}{{H}_{3}}$$

Similarly, the ${d}_{31}^{*}$ values of the CNF–CoFe_2_O_4_ composite papers containing 10.9, 21.0, and 27.5 vol% CoFe_2_O_4_ were calculated as 0.391 $\times$ 10^−12^, 18.8 $\times$ 10^−12^, and 27.1 $\times$ 10^−12^ m/A, respectively.

**Figure 8:**
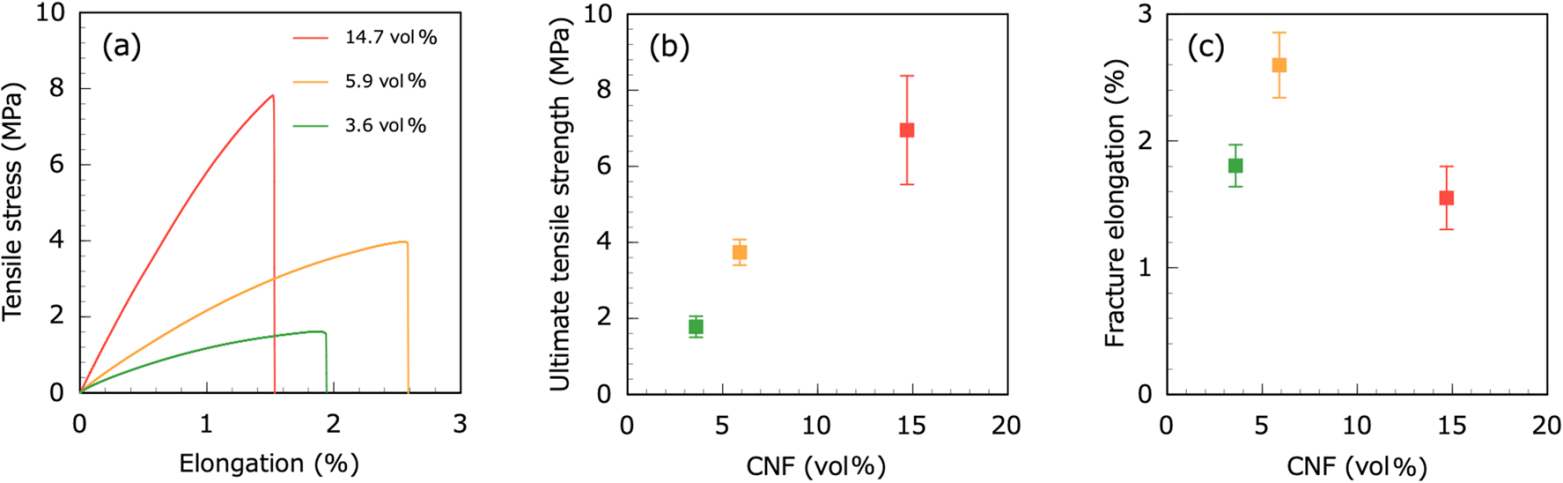
(**a**) Stress-elongation curves of the CNF–CoFe_2_O_4_ composite papers; plots of (**b**) UTS and (**c**) fracture elongation versus CNF volume fraction in the CNF–CoFe_2_O_4_ composite papers.

Figure 8a shows the stress-elongation curves of the CNF–CoFe_2_O_4_ composite papers. Here, the elongation was estimated from the displacement of the universal testing machine crosshead. The initial slopes (between 0 and 0.2% elongation) of the stress–elongation curves of the CNF–CoFe_2_O_4_ composite papers were calculated for the 10.9, 21.0, and 27.5 vol% specimens, and they were determined as 0.523, 0.269, and 0.195 GPa, respectively (see Table 1). These values were taken as the apparent effective Young’s moduli. Figures 8 (b and c) plot the ultimate tensile strengths (UTSs) and fracture elongations versus CNF volume fraction in the CNF–CoFe_2_O_4_ composite papers. The CNF volume fractions in the 10.9, 21.0, and 27.5 vol% composite papers were 14.7, 5.9, and 3.6 vol%, respectively. The UTS of the CNF–CoFe_2_O_4_ composite paper was increased by the addition of CNFs. However, the addition of CoFe_2_O_4_ particles decreased the UTS, i.e., CNF was responsible for the tensile properties of CNF–CoFe_2_O_4_ composite papers. Therefore, the magnetic and magnetostrictive properties and tensile properties of CNF–CoFe_2_O_4_ composite papers can be controlled by changing the mixture ratio of CNF and CoFe_2_O_4_ particles. The apparent ${k}_{33}^{2}$ values of the 10.9, 21.0, and 27.5 vol% CNF–CoFe_2_O_4_ composite paper were 5.45 $\times$ 10^−16^, 9.37 $\times$ 10^−15^, and 2.36 $\times$ 10^−14^, respectively (see Table 1). The improved magneto-mechanical coupling factor after adding CoFe_2_O_4_ implies that the CNF–CoFe_2_O_4_ composite paper is a promising candidate for energy harvesting applications.

## Conclusion

This study evaluated the magnetic, magnetostrictive, and tensile properties of CNF–CoFe_2_O_4_ composite papers with different volume fractions of CoFe_2_O_4_. The XRD and EDX analyses revealed that CoFe_2_O_4_ remained stable during the fabrication process. The SEM images confirmed that CoFe_2_O_4_ particles were dispersed through the CNF matrix but were sometimes agglomerated. The CoFe_2_O_4_ particles imparted magnetization to the CNF paper and the maximum magnetization of the CNF–CoFe_2_O_4_ composite paper was a linearly increasing function of CoFe_2_O_4_ content. The magnetostriction of the CNF–CoFe_2_O_4_ composite paper was negative and positive in the directions parallel and perpendicular to the magnetic field, respectively. The apparent effective Young’s modulus and UTS of the CNF–CoFe_2_O_4_ composite paper decreased with increasing CoFe_2_O_4_. This was because increased amount of CoFe_2_O_4_ particles decreased the CNF volume fraction of the composite paper. Conclusively, the CNF was responsible for the tensile properties of CNF–CoFe_2_O_4_ composite paper. Therefore, the magnetic and magnetostrictive properties and tensile properties of CNF–CoFe_2_O_4_ composite paper can be controlled by changing the mixture ratio of CNF and CoFe_2_O_4_ particles. Overall, the CoFe_2_O_4_ additives impart magnetic and magnetostrictive properties to the CNF paper and may increase its toughness in exchange for decreasing its tensile properties. The magneto-mechanical coupling factor of the paper was improved by adding CoFe_2_O_4_ particles; therefore, the CNF–CoFe_2_O_4_ composite paper is expected to be available for energy harvesting applications.

## Data Availability

The materials described in the manuscript, including all relevant raw data, will be freely available to any researcher wishing to use them for non-commercial purposes from the corresponding author on reasonable request.
